# Formeln an der Arbeit: Technische Spezialrechenschieber aus der Sammlung des Deutschen Museums

**DOI:** 10.1007/s00048-025-00412-w

**Published:** 2025-03-19

**Authors:** Hannes Junker

**Affiliations:** Mathematisches Institut, Universität Bonn, Endenicher Allee 60, 53115 Bonn, Deutschland

**Keywords:** Rechenschieber, Technische Mathematik, Rationalisierung, Betriebsrechnung, Extraktausbeute, Schiffsdimensionierung, Slide rules, Technical mathematics, Scientific management, Brewing, Shipconstruction

## Abstract

Das Deutsche Museum in München verfügt über eine umfangreiche Sammlung an Recheninstrumenten. Den Hauptbestand bilden mechanische Rechenschieber und -scheiben verschiedener Hersteller der Jahre 1870 bis 1970. Zu diesen Geräten gehört auch eine größere Anzahl an Spezialrechenschiebern aus technischen Bereichen, die den Ausgangspunkt für ein Forschungsvorhaben am Museum bildeten. Der Artikel erschließt den professionellen Entstehungs- und Verwendungskontext ausgewählter Objekte aus der Betriebsrechnung, dem Brauwesen und dem Schiffbau. Vor diesem Hintergrund werden technische, ökonomische und soziale Faktoren herausgearbeitet, die die Einführung mathematischer Methoden in die jeweiligen Arbeitsfelder förderten oder hemmten. Abschließend wird anhand der Beispiele die Rolle der Mathematik bei der Umgestaltung technischer Gewerbe und Berufe während der Hochindustrialisierung diskutiert.

## Abstract (en)

The Deutsches Museum in Munich has an extensive collection of calculating devices. The main collection consists of mechanical slide rules from various manufacturers from the years 1870–1970. This includes a large number of special slide rules from technical fields, which formed the starting point for a research project at the museum. The article explores the professional context of selected objects which were used in scientific management, brewing and shipbuilding. Against this background, technical, economic and social factors promoting or inhibiting the integration of mathematical methods in the respective fields of work are identified. Finally, the examples are used to discuss the role of mathematics in the transformation of technical trades and professions during the period of high industrialization.

## Einleitung

Die industrielle Herstellung der Rechenschieber begann in Deutschland nach der Gründung des Kaiserreichs. Während des Deutsch-Französischen Krieges brachen die Importe aus Frankreich, das zu jener Zeit eine führende Stellung bei der Produktion der Instrumente innehatte, ein. Diese Lücke wurde nach und nach von deutschen Firmen wie Dennert & Pape, Faber-Castell und Albert Nestler Zeichentechnik geschlossen. Bis zur Jahrhundertwende weitete sich die Produktion von Rechenschiebern im Deutschen Kaiserreich aus. Die fortschreitende Industrialisierung ließ die Nachfrage nach mathematischen Instrumenten in vielen Wirtschaftszweigen wachsen, schuf aber auch einen Markt für Spezialanfertigungen, die bei wiederkehrenden Berechnungen Verwendung fanden.

Die Geschichte der Rechenschieber ist eng verwoben mit den Transformationsprozessen, welche in den Jahren 1871 bis 1930 für eine tiefgreifende Umgestaltung der Arbeit in vielen Gewerben sorgten. Die Anwendung wissenschaftlicher Methoden veränderte die Arbeitsweise in den Betriebs- und Fabrikhallen, aber auch in den Büros der Unternehmer. Traditionelle Formen der Produktion, die durch Handwerkstechniken geprägt waren, wurden verdrängt. Die neuen Methoden erforderten genaue Messungen. Das Gespür für einzelne Arbeitsschritte, das Arbeiter und Inhaber über Jahre hinweg verfeinerten, aber auch Berufserfahrung im allgemeineren Sinne büßten bei der Ausübung beruflicher Tätigkeiten zunehmend an Bedeutung ein. Neben Maschinen, die nun in vielen Fabriken den Takt vorgaben, prägten Formeln diesen vielschichtigen Prozess (Fleischman & Tyson 1993; Kelly & Gráda 2022).

Einerseits bildete die Anwendung mathematischer Methoden ein wichtiges Moment bei der Rationalisierung der Betriebsführung und der Standardisierung von Fertigungsprozessen (Gasca et al. 2012). Das Wachstum der Unternehmen, angeregt durch die Konzentrationsprozesse während der Industrialisierung, erschwerte zunehmend die Geschäftsleitung. Die große Anzahl an Angestellten und die hohen Anschaffungskosten für Maschinen trugen ebenfalls dazu bei, den Planungshorizont auszudehnen. Methoden des Managements fassten dadurch während der Hochindustrialisierung auch in Deutschland Fuß (Chandler 1977). Im Zuge dessen veränderten sich Buchführung und Geschäftsleitung in den Betrieben. Die Inhaber griffen zunehmend auf Statistiken zurück, um sich einen Überblick über die Entwicklungen in den einzelnen Bereichen des Unternehmens zu verschaffen (Porter 1995). Unterdessen riefen die neuen Formen der Massenfertigung auch Änderungen in den Konstruktionsbüros hervor. Die serielle Fertigung in großen Stückzahlen verlangte einerseits eine präzise Planung der technischen Teile, andererseits traten bei der Skalierung technischer Anlagen Probleme auf, die eine theoretische Durchdringung erforderten (Heun 1901). Auch der Ingenieur, der in den Werften und Betrieben für die Konstruktion neuer Anlagen Verantwortung trug, konnte immer weniger auf seine Erfahrung zurückgreifen, sondern musste sich bei der Arbeit verstärkt mathematischen Methoden aus dem Gebiet der technischen Mechanik anvertrauen.

Die Geschichte dieser Umwälzung wurde bereits aus verschiedenen Perspektiven erzählt. Im Fokus standen oft die technischen Innovationen, die die Produktion prägten, Veränderungen bei der Betriebsstruktur und Arbeitsorganisation, aber auch die gesellschaftlichen Verschiebungen infolge der Industrialisierung. Zwar entging der Forschung nicht, welchen Anteil hierbei die Integration mathematischer Methoden in die Arbeitswelt besaß, doch es fehlen nach wie vor eigenständige Untersuchungen zu diesem Thema. Die Gründe für diese Leerstelle lassen sich auf zwei Aspekte zurückführen: Die Historiografie der Mathematik brachte dem Thema wenig Beachtung entgegen, da es abseits der akademischen Tradition liegt, die immer noch im Mittelpunkt der Forschung steht. Innerhalb der Technikgeschichte wird der Anwendung mathematischer Methoden unterdessen als Aspekt bei der Einführung wissenschaftlicher Methoden zwar Bedeutung beigemessen, jedoch wird sie selten problematisiert. Sie erscheint stattdessen als eine Begleiterscheinung im Zuge eines allgemeinen Prozesses, der durch technologische Innovationen und ökonomische Umwälzungen gekennzeichnet ist.

Dadurch bleiben Fragen unbeantwortet, die längst nicht nur für die Geschichte der Mathematik von Interesse sind. Die Einbindung mathematischer Methoden in die Arbeitsprozesse, die sich nach 1871 in vielen Bereichen vollzog, hatte auch eine soziale Dimension. Formeln halfen nicht nur, technische Probleme zu lösen – und ebenso wenig erschöpft sich ihre praktische Bedeutung in der bloßen Anwendung von Konzepten, wie es zuweilen aus der Perspektive der modernen Mathematik erscheint. Mathematische Methoden veränderten, wo sie in den Arbeitsprozess integriert wurden, die Tätigkeit stattdessen in einem umfassenden Sinne. Über Formeln wurden im professionellen Zusammenhang Normen formuliert und Regeln durchgesetzt, aber auch Arbeitsleistung gemessen und Sollwerte aufgestellt (Daston 2022). Mathematik änderte nicht nur die technischen Parameter der Produktion, sondern griff auch unmittelbar in die Tätigkeit der Menschen ein. Sie prägte die Lebenserfahrung um 1900, was sich auch in der Literatur niederschlug. Der Erzähler in Upton Sinclairs Roman *Der Dschungel* sprach mit Blick auf die gewaltigen Schlachthöfe in Chicago von „Schweinefleischfabrikation mit angewandter Mathematik“ (Sinclair 1906). Und spürten nicht die Bergarbeiter in Émile Zolas *Germinal *genau, dass die neue Formel zur Bestimmung des Akkordlohns sie endgültig ins Verderben stürzen wird (Zola 1885)? Ihnen wurde vor Augen geführt, dass ihr eigenes Schicksal an Rechenvorschriften hing. Ihre Arbeit, aber auch die der Bergwerksleitung wurde immer mehr von Zahlen und Arithmetik beherrscht.

### Mathematische Instrumente

Dieser Prozess ging im Lärm der Industrialisierung unter. Während sich das Hämmern und Schnauben der Maschinen noch aus der Ferne wahrnehmen lässt, fanden mathematische Methoden leise ihren Weg in die Arbeitswelt. Dennoch hinterließ ihre Integration auf lokaler Ebene Zeugnisse, die heute als Ausgangspunkt für historiografische Studien über Mathematik in der Arbeitswelt gelten können. Für viele Aufgaben wurden spezielle Rechenhilfen entwickelt. Wegweisend für die Anwendung mathematischer Methoden im professionellen Kontext waren Instrumente und Grafiken aller Art (Gessner et al. 2018; Kleine 2017). Diagramme erleichterten Forschern und Unternehmern die Interpretation statistischer Daten (Friendly & Wainer 2021; Hankins 1999; Holmes & Olesko 1995). Den Ingenieuren stand neben Tabellen- und Tafelwerken auch eine Vielzahl mechanischer Instrumente zur Verfügung – darunter beispielsweise Geräte zur Integration (Polarplanimeter, Integrafen) (Tobies 2013). Eine außerordentliche Bedeutung besaßen spezielle Rechenschieber, die eine einfache Auswertung mathematischer Formeln ermöglichten (Kidwell 2022, 2015). Sie fanden vor allem im Ingenieurswesen große Verbreitung.

An der Konstruktion neuer Instrumente und Grafiken beteiligten sich auch deutsche Mathematiker wie Carl Runge und Rudolf Mehmke (Runge 1909; Mehmke 1890).

Die meisten Spezialrechenschieber wurden allerdings von Ingenieuren und Wissenschaftlern entworfen, wie auf den folgenden Seiten deutlich werden wird. Durch die Konstruktion versuchten sie häufig die Bearbeitung wiederkehrender Aufgaben zu erleichtern, die im Umfeld ihrer Tätigkeit auftraten. Nicht selten wurden Spezialrechenschieber in der Absicht entworfen, die Anwendung mathematischer Methoden zu vereinfachen und hierdurch ihre Verbreitung in bestimmten Arbeitsfeldern voranzutreiben. In Zeiten, in denen das allgemeine Niveau der mathematischen Bildung noch niedrig war, bildeten umfangreiche Berechnungen einen hemmenden Faktor bei der Anwendung neuer Verfahren. Wer an der Etablierung mathematischer Verfahrensweisen interessiert war, musste deshalb Wege finden, den rechnerischen Aufwand zu verringern. Die Anfertigung angepasster Rechenschieber erwies sich hierfür als geeignetes Mittel.

Nicht zuletzt deshalb bilden die Objekte einen hervorragenden Ausgangspunkt, um die Integration mathematischer Methoden in technische Tätigkeitsfelder zu untersuchen. In diesem Artikel werden Spezialrechenschieber aus der Sammlung des Deutschen Museums vorgestellt. Die ausgewählten Geräte der Zeit 1870 bis 1930 stammen aus drei technischen Bereichen: der Betriebsrechnung, dem Brauwesen und dem Schiffbau. Im Hauptteil des Artikels wird die Entstehungsgeschichte dieser Rechenschieber erschlossen. Das Augenmerk liegt einerseits auf den Problemen, die in den jeweiligen Gebieten zur Konstruktion der Geräte Anlass gaben. Andererseits werden die spezifischen Faktoren herausgearbeitet, welche in die mathematische Konzeption der Rechenschieber hineinwirkten. Vorweg werden die historischen Hintergründe der Münchener Museumssammlung und die allgemeine Bedeutung von Spezialrechenschiebern erläutert.

### Die Firma Dennert & Pape

Die Sammlung an Rechenschiebern des Deutschen Museums stammt in großen Teilen aus dem Nachlass der Firma Dennert & Pape, der 2002 nach München kam. Die Geschichte des Unternehmens umspannt die Jahre 1862 bis 1978. Nach dem Ende des Deutsch-Französischen Krieges begann die Hamburger Firma mit der Herstellung von Rechenschiebern (Dennert & Pape Aristo-Werke 1962). Während der Hochindustrialisierung wuchs das Geschäft mit den Instrumenten stetig an. In den 1880er Jahren lief die Fertigung von Sonderrechenschiebern für die Landesvermessung an. Diese Instrumente wiesen gegenüber dem allgemeinen Rechenstab eine besondere Skaleneinteilung auf, die für die geodätischen Berechnungen angepasst war. Im Laufe der Zeit erweiterten die Inhaber von Dennert & Pape das Sortiment an Spezialrechenschiebern. Die Geräte wurden teils im Auftrag produziert, teils auch für den Markt entworfen. Um 1900 gehörte das Unternehmen schließlich zu den größten Produzenten von Rechenschiebern im Deutschen Reich.

Nach der Jahrhundertwende vertrieb Dennert & Pape eine breite Palette von Geräten unter den Markennamen Dupa (ab 1924) und Aristo (ab 1936) im In- und Ausland (Dennert 1997). Während der beiden Weltkriege lief die Produktion von Rechenschiebern weiter. Reichswehr und Wehrmacht bestellten in großer Stückzahl Instrumente bei dem Unternehmen, darunter vor allem verschiedene Typen von Artillerie- und Navigationsrechenschiebern.^1^ Mit dem Ende des Zweiten Weltkriegs richtete Dennert & Pape die Produktion wieder auf den zivilen Markt aus. In den Klassenräumen lernten die Schüler an Aristo-Instrumenten die Bedienung von Rechenschiebern, während Industrieunternehmen neue Spezialanfertigungen bei der Firma in Auftrag gaben. In den 1970er Jahren leitete dann die großflächige Markteinführung des elektronischen Taschenrechners das Ende der Rechenschieberproduktion ein. Im Jahr 1978 wurde die Herstellung der Instrumente eingestellt.

Die Sammlung des Deutschen Museums spiegelt nicht nur die Geschichte der Firma wieder. Die Inhaber von Dennert & Pape sammelten auch systematisch Produkte der internationalen Konkurrenz. Im Depot des Museums lagern deshalb auch zahlreiche Erzeugnisse anderer Firmen, die während der Jahre 1872 bis 1978 gemeinsam mit den Produkten des Hamburger Unternehmens am Markt für Recheninstrumente präsent waren. Überdies umfasst der Nachlass neben Firmenschriften auch Auszüge aus Gebrauchsmustern, Patenten und Werbeschriften für Rechenschieber verschiedener Entwickler und Hersteller. Gemeinsam mit den Objekten bildeten sie wichtige Quellen für den vorliegenden Artikel, in dem Spezialrechenschieber aus unterschiedlichen Berufs- und Tätigkeitskontexten vorgestellt werden.

### Spezialrechenschieber

Gewöhnliche Rechenschieber leisteten lange Zeit gute Dienste bei der Rechnung mit Stift und Papier, indem sie bei der Auflösung arithmetischer Aufgaben halfen. Die Instrumente funktionieren nach dem Prinzip der grafischen Addition. Durch das Herausziehen einer Zunge aus einem Korpus werden zwei parallele Skalen gegeneinander verschoben. Wird die Zunge um eine feste Strecke a herausgezogen, so lässt sich gegenüber von dem Wert b der Skala auf dem Körper die Zahl a + b auf der Zunge ablesen, wenn die beiden Teilungen gleichmäßig sind. Die gewöhnlichen Rechenschieber weisen logarithmische Skalenteilungen auf, wodurch sie sich auf Grundlage desselben Prinzips für die grafische Auflösung von Multiplikations- und Divisionsaufgaben verwenden lassen.

Im Gegensatz zu diesen arithmetischen Rechenhilfsmitteln sind Spezialrechenschieber für die Auswertung spezifischer Funktionen konstruiert worden. Ihre Skalen wurden für den jeweiligen Zweck angepasst. In der Berufspraxis – vor allem im technischen Bereich – fanden sie bei der Auflösung wiederkehrender Aufgaben Verwendung. Mit den Geräten ließen sich gesuchte Werte, Kennzahlen und Normgrößen durch wenige Handgriffe ermitteln. Die angepasste Konstruktion erübrigte in der Regel jede weitere Berechnung per Hand.

Nicht zuletzt aufgrund ihrer leichten Bedienbarkeit boten sich Spezialrechenschieber als Alternative zu Tabellen und grafischen Tafeln an. Aufgrund ihrer handlichen Größe besaßen sie außerdem Vorzüge bei der Arbeit unter freiem Himmel, weswegen sie in der Armee und bei Ingenieuren größere Verbreitung fanden. Neben den Vorteilen beim praktischen Gebrauch gab es jedoch auch technische Gründe, die für Spezialrechenschieber sprachen. Die Anfertigung eines Tabellenwerks kommt nur für Funktionen infrage, die von höchstens drei Größen abhängen. Während der Wert einer Größe durch die Buchseite mit der entsprechenden Tabelle vorgegeben wird, werden die anderen beiden Größen über die Zeile und Spalte bestimmt, in deren Schnittpunkt sich dann der gesuchte Funktionswert ablesen lässt. Im technischen Zusammenhang tritt jedoch häufig eine größere Zahl an Parametern auf. Für die Bestimmung des Abschusswinkels bei der Artillerie spielten etwa die Windrichtung und -stärke, Entfernung und Richtung zum Ziel, aber auch der Luftdruck eine nicht unwesentliche Rolle.

Für derartige Anwendungen empfahl sich die Konstruktion eines passenden Rechenschiebers. Zwar erfordert jede Veränderliche eine eigene Skala, aber die parallele Anordnung der Skalen auf den Zungen erleichtert es dem Benutzenden, die Übersicht zu behalten.

Die konzeptionellen Vorteile führten zu einer großflächigen Verbreitung von Spezialrechenschiebern während der Hochindustrialisierung. Die handlichen Geräte halfen bei der Integration mathematischer Methoden in Betriebsabläufe und Arbeitsfelder. Frederick W. Taylor (1856–1915) erkannte früh den Wert, den Rechenschieber darüber hinaus als Kontrollinstrument in den Händen von Betriebsleitern besaßen.

## Schnittanzeiger

**Abb. 1:**
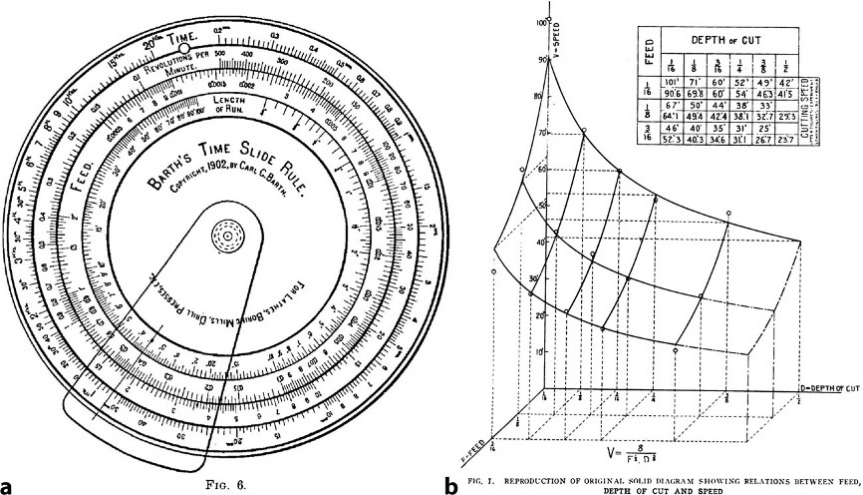
**a** Darstellung einer Rechenscheibe, die während Taylors Untersuchungen entstanden ist. Der Entwurf stammt von seinem Mitarbeiter Karl Georg Barth (Barth 1903: 59). **b** Reproduktion eines Plots, mit dem Barth Messdaten approximierte, die Taylors Team gesammelt hatte. Er veranschaulicht den Zusammenhang zwischen Vorschub, Schnitttiefe und Geschwindigkeit einer Drehbank (Barth 1919: 173)

In den Jahren 1880 bis 1907 hat der US-amerikanische Ingenieur mit einem Team von Mitarbeitern Untersuchungen an Drehbänken und Fräsmaschinen in US-amerikanischen Stahlbetrieben durchgeführt. Die Ergebnisse fasst ein Bericht zusammen, der unter dem Titel „On the Art of Cutting Metals“ in der Zeitschrift der American Society of Mechanical Engineers erschienen ist. Die umfangreiche Abhandlung endet mit der Besprechung eines kreisrunden Rechenschiebers, der während der gemeinsamen Arbeit entstanden war. Für Frederick W. Taylor war das unscheinbare Gerät das wichtigste Ergebnis der jahrelangen Forschung (Abb. 1).

Mit den Untersuchungen verfolgte Taylor das Ziel, die Einstellung von Drehmaschinen zu optimieren, um für verschiedene Konfigurationen die jeweils optimale Schnittgeschwindigkeit zu erhalten. Für jede Art von Werkstück und -zeug wollte er die beste Maschineneinstellung finden, die den Verschleiß und die Bearbeitungszeit minimiert. Über Jahre maß das Team den Einfluss einzelner technischer Parameter auf die Laufzeiten der Drehmaschinen. In den Messreihen untersuchten sie insgesamt zwölf Faktoren (Taylor 1907). Um den Einfluss eines Parameters zu ermitteln, variierten sie ihn von Messung zu Messung, während sie alle anderen Größen konstant hielten. Mit dieser mühsamen Arbeit verband Taylor handfeste Absichten. Er wollte die Ausnutzung der vorhandenen Ressourcen in den Betrieben verbessern.

Auf Grundlage der Untersuchungen entstanden mehrere Rechenschieber, mit denen sich für verschiedene Materialstärken und Werkzeugformen jeweils die optimale Einstellung für die Drehmaschinen bestimmen ließ. Taylor hielt sie für die größte Errungenschaft seiner langjährigen Untersuchungen. In seinen Augen ermächtigten die Geräte das Management, die Arbeit in den Betrieben nach wissenschaftlichen Gesichtspunkten zu organisieren. Die Betriebsleiter erhielten Kontrolle über die Arbeit in den Werkshallen:

„The gain from these slide rules is far greater than that of all the other improvements combined, because it accomplishes the original object, for which in 1880 the experiments were started; i.e. that of taking the control of the machine shop out of the hands of the many workmen, and placing it completely in the hands of the management, thus superseding ‚rule of thumbs‘ by scientific control“ (Taylor 1907: 39f.).

### Mathematische Hürden

Die Bestimmung der optimalen Werte für die einzelnen Maschinen erwies sich allerdings als hartnäckiges Problem. Taylor und seine Mitarbeiter hatten über Jahre hinweg Seite um Seite an Messdaten angehäuft. Ihnen gelang es jedoch nicht, aus diesen Werten eine Formel herzuleiten, mit der sich die ideale Maschineneinstellung finden ließ. Aufgrund der großen Anzahl involvierter Faktoren geriet die Arbeit zwischenzeitlich ins Stocken. Selbst US-amerikanische Mathematiker, die Taylor um Rat bat, wussten keine Lösung für sein Problem (Taylor 1907: 40).

Ein Durchbruch gelang erst dem Norweger Karl Georg Barth (1860–1939), der sich 1899 Taylors Gruppe anschloss. In nur wenigen Monaten gelang es Barth, eine passende Formel herzuleiten. In einem mehrteiligen Artikel für *Industrial Management*, der 1919 erschien, als Taylor bereits verstorben war, berichtet Barth rückblickend von seinem Vorgehen (Barth 1919). Er verwendete Methoden der Ausgleichsrechnung, um zwischen den gemessenen Werten einen funktionalen Zusammenhang herzustellen. Sein Artikel enthält die Grafik einer Fläche, mit der er die Messdaten interpoliert hat (Abb. 1). Daneben präsentiert er eine Gleichung,

$$V=\frac{8}{F^{1/2}D^{3/8}}$$

welche eine Relation zwischen der Schnittgeschwindigkeit V, dem Vorschub F und der Schnitttiefe D herstellt (Barth 1919: 173).

In seinem Bericht erklärt Taylor, Barth sei der beste Mathematiker in seinem Team gewesen (Taylor 1907: 35). Zwischenzeitlich war zwischen beiden jedoch ein Streit über das weitere Vorgehen entbrannt. Für die Herleitung der Formel hatte sich Barth zuerst auf die wichtigsten Parameter konzentriert, damit das Ergebnis übersichtlich blieb. Taylor drängte indessen darauf, möglichst viele technische Parameter zu berücksichtigen, um eine genaue Abbildung der tatsächlichen Verhältnisse zu erhalten. Je mehr Variablen einflossen, desto verwickelter wurde jedoch der mathematische Ausdruck. Gegenüber Taylor konnte sich Barth mit seinen Bedenken nicht durchsetzen. In dem Abschlussbericht „On the Art of Cutting Metals“ findet sich stattdessen die Formel

$$V=C\cdot \frac{1-\frac{8}{7\left(32r\right)^{2}}}{F^{\frac{2}{5}+\frac{2.12}{5+32r}}\cdot \frac{48}{32r}\cdot D^{\frac{2}{15}+0.06\sqrt{32r}+\frac{0.8\left(32r\right)}{6\left(32r\right)+48D}}}$$

für die Berechnung der Schnittgeschwindigkeit V, die zusätzlich noch vom Radius r des Werkzeugs abhängt (Taylor 1907: 204). Die Konstante C ist ein Erfahrungskoeffizient, der sich nach der Härte des Materials und der Qualität des Werkzeugs richtet.

### Betriebsrechenschieber System Friedrich und Hippler

Es dauerte einige Jahre, bis Taylors Theorien des „Scientific Managements“ in Deutschland großflächig Verbreitung fanden. Spätestens um 1920, nach dem Ende des Ersten Weltkriegs, waren Fach- und Berufszeitschriften jedoch voll von Artikeln zur „rationellen Betriebsführung“. Allerorten wurden nun Untersuchungen an Drehbänken und Fräsmaschinen unternommen. Zeitgleich entstanden viele Initiativen, die die Verwendung von Rechenschiebern in Betrieben propagierten (Overmann 1922).

**Abb. 2:**
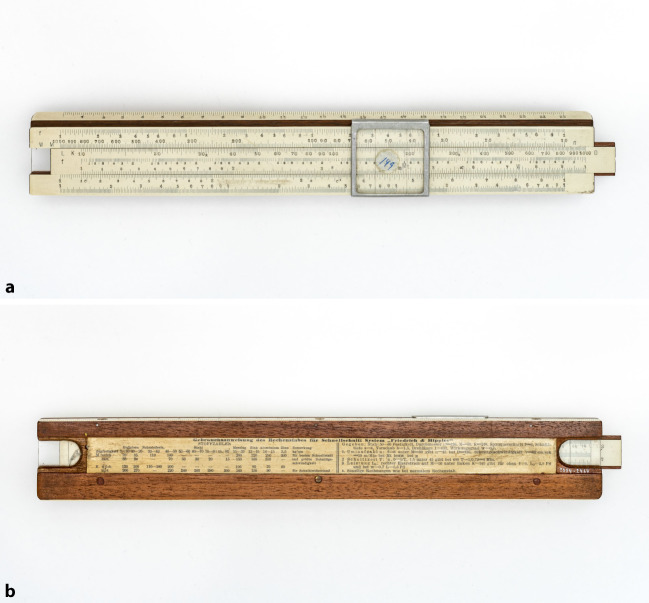
Vorderseite (**a**) und Rückseite (**b**) eines Betriebsrechenschiebers des Systems Friedrich-Hippler, 1928 (Deutsches Museum München, Inv.-Nr. 2004-2467)

Auch Dennert & Pape brachte während dieser Zeit verschiedene Geräte auf den Markt. Eines der ersten Geräte im Sortiment war der „Betriebsrechenschieber System Friedrich & Hippler“ (Abb. 2), das 1925 auf den Markt kam.^2^ Die Hamburger Firma reagierte damit auf das Interesse, das die Arbeit Taylors in Deutschland hervorgerufen hatte. In einer Werbeschrift von Dennert & Pape heißt es: „Er ersetzt den Taylorschen Rechenschieber in der deutschen Werkstatt und übertrifft ihn an Einfachheit in der Ausführung.“^3^

Willy Hippler, einer der beiden Namensgeber des Betriebsrechenschiebers, war als Dozent an der Technischen Hochschule in Breslau tätig. In einem Lehrbuch zur Betriebsführung in Werkstätten stellte er ein Gerät vor, das 1914 ein Dozent an der Technischen Staatslehranstalt in Chemnitz entwickelt hatte (Hippler 1919: 42ff.). Der Ingenieur H. Friedrich hatte in den Jahren 1909 bis 1914 eine Reihe von Untersuchungen in der Maschinenlehrausstellung der Königlich Technischen Hochschule Dresden an einer Schnelldrehbank durchgeführt. Die Ergebnisse präsentierte er in einem längeren Artikel, an dessen Ende er auch seinen „Schnittanzeiger“ vorstellte. Hippler empfahl den Friedrichschen Schnittanzeiger für die Verwendung, stellte aber an derselben Stelle eine verbesserte Ausführung vor, die er selbst angefertigt hatte. In seinem Buch führt er das Grundproblem aus, das die Einführung spezieller Rechenschieber erforderlich mache: „Dem Arbeiter tritt täglich, wenn er ein neues Stück auf die Bank nimmt, die Frage entgegen: mit welcher Schnittgeschwindigkeit soll ich die Maschine laufen lassen, welchen Span soll ich nehmen und welche Form muss der Stahl haben?“ (ebd.: 8ff.)

Auf diese Fragen gab der Rechenschieber eine Antwort. Auf der Vorder- und Rückseite des herausnehmbaren Schiebers und auf dem Rahmen des Geräts sind mehrere Skalen zu technischen Parametern angebracht. Wie es in dem Katalog von Dennert & Pape heißt, ließen sich mit dem Gerät die „günstigsten Werte für die Schnittgeschwindigkeit, Vorschub und Schnitttiefe bei Naß- und Trockenbearbeitung verschiedener Metalle“ bestimmen.^4^ Für die Bestimmung der Schnittgeschwindigkeit v und der Umlaufzahl *n* wird auf der Rückseite des Rechenschiebers ein Beispiel gegeben. Die Zunge wird verschoben, bis der gegebene Spanquerschnitt f = 6 (Schieber) unter der Materialkonstante M = 50 (Oberer Rahmen) steht. Gegenüber des Durchmessers D = 136 in Millimetern des Werkstücks, der auf der Zunge abgetragen ist, lässt sich dann die gesuchte Umlaufzahl *n* = 45 bestimmen. Oberhalb von der Markierung M_t_ für Trockenschnitt steht dann die Schnittgeschwindigkeit v = 36 in Zentimeter pro Sekunde, mit der die Drehbank laufen sollte.

Mit dem Rechenschieber ließ sich nicht nur eine Einstellung für die Maschine finden, die zumindest der Idee nach optimale Ergebnisse versprach, sondern auch vorab die Arbeitszeit ermitteln, die für die Bearbeitung eines Werkstücks fester Länge bei dieser Einstellung anfiel. Diese Kalkulation war vor allem für die Betriebsführung von Interesse. Sie konnte hiernach nicht nur die Arbeitszeit einzelner Arbeitsschritte vorab bemessen, sondern auch die Kosten veranschlagen. In den Händen der Betriebsleiter wurde das Gerät auch zu einem Kontrollinstrument, um die Arbeiter an den Maschinen im Blick zu behalten.

### Normalisierung

Allerdings war auch Hippler nicht nur daran gelegen, die Maschinisten bei ihrer Tätigkeit zu unterstützen. Um die Widerstände zu überwinden, die der Einführung wissenschaftlicher Methoden im Weg standen, weist er auf den großen Anteil der Lohnkosten an der Produktion hin (ebd.: 31). Für die Festsetzung des Akkordlohns müsse die Leistungsfähigkeit einer Drehbank bestimmt werden, aber auch die wirtschaftlichste Schrittgeschwindigkeit für jedes Gerät in Abhängigkeit vom Material, Durchmesser und der jeweiligen Operation, die an dem Werkstück ausgeführt werden soll. Auf dieser Grundlage müssten dann Normen entwickelt werden, nach denen sich der Vorarbeiter richten muss.

Nachdem er die Funktionsweise des Geräts erläutert hat, kommt Hippler auf eine zentrale Schwäche des Entwurfs zu sprechen: Friedrich hatte seine Formeln an einer bestimmten Drehbank entwickelt. Für die Arbeit an anderen Maschinen gab es keine Garantie, dass der Rechenschieber auch optimale Ergebnisse liefert (ebd.: 48). Hippler erwähnt zwar, dass der Taylorsche Rechenschieber diesen Nachteil nicht besitzt.^5^ Sogleich fügt er jedoch an, dass die „Normalisierung der Werkzeugmaschinen“ das Ziel der Branche sein müsse, damit alle Maschinen gleiche Vorschübe und Schnittgeschwindigkeiten liefern. Das Hauptproblem war in seinen Augen die fehlende Standardisierung.

Die beschränkte Anwendungsmöglichkeit von Hipplers Rechenschieber blieb nicht unbemerkt. In einer Besprechung für die Zeitschrift *Werkstattstechnik* aus dem Jahr 1920 heißt es, dass sich die Anschaffung aufgrund des hohen Preises (180 Mark) nur für Betriebe rechtfertige, die eine passende Ausstattung besäßen (Anonym 1920). Dennert & Pape bot das Gerät einige Jahre später für 280 Mark an, wie aus der Werbeschrift hervorgeht.^6^ Offenbar war es auch Hippler nicht gelungen, das Grundproblem, dass die Gültigkeit der Rechenschieber von den spezifischen Eigenschaften der Drehbänke abhängt, zu lösen. In einem anderen Artikel, der zwei Jahre später in derselben Zeitschrift erschien, wird das Problem erneut angesprochen. Der Autor des Textes erklärt, dass Hipplers Rechenschieber zu hohe Werte liefere, die nur bei erstklassigem Material und Hochleistungsarbeitsmaschinen erreicht würden (Theimer 1922).

Womöglich auch aufgrund dieser Kritik brachte Dennert & Pape wenige Jahre später, 1927, einen neuen Rechenschieber heraus. Mit dem Betriebsrechenschieber System Kresta konnten, wie es in einer Werbeschrift heißt, alle für den Betrieb von Drehbänken und Fräsmaschinen unerlässlichen Werte bestimmt werden.^7^ Während er in dieser Hinsicht dem Vorgängermodell Marke Hippler und Friedrich in nichts nachstand, wurde mit Nachdruck darauf hingewiesen: „Die ermittelten Werte sind praktisch erprobt und können in jedem normalen Betrieb *zuverlässig* eingehalten werden.“ (Auszeichnung übernommen)

Taylor wollte nicht nur die Laufzeiten der Maschinen mithilfe mathematischer Methoden optimieren, sondern die Rechenschieber sollten auch dabei helfen, die Tätigkeit der Arbeiter an Regeln zu binden. In diesem Sinne war Mathematik ein wichtiges Instrument bei der Durchsetzung von Normen im Arbeitsumfeld. Gleichzeitig erwies sich die fehlende Standardisierung bei den Drehbänken jedoch als Hindernis hierfür. Das große Spektrum an Maschinen, aber auch die Vielzahl an technischen Parametern erschwerten die Entwicklung allgemeingültiger Formeln.

Trotz der technischen Hürden blieben Rechenschieber bei der Betriebsrechnung noch lange Zeit in Gebrauch. Seit dem Ende des Ersten Weltkriegs förderte der Ausschuss für wirtschaftliche Fertigung (AWF) die Konstruktion von Rechenhilfen. Ab 1922 dem Reichskuratorium für Wirtschaftlichkeit in Industrie und Handwerk untergeordnet, brachte der Ausschuss Spezialrechenschieber für die Betriebs- und Konstruktionsrechnung auf den Markt – darunter auch Instrumente für die Kalkulation von Schnittgeschwindigkeiten (Bahlecke 1931; Greis 2021). Während der Vertrieb dieser Geräte bis in die Nachkriegszeit andauerte, trug die fortschreitende Automatisierung technischer Maschinen und Anlagen dazu bei, die Recheninstrumente aus den Betrieben zu verdrängen.

## Malzausbeute

Noch Mitte des 19. Jahrhunderts glich das Bierbrauen einem Mysterium. Von den chemischen Prozessen, die in den Sudhäusern und Gärkellern stattfinden, verstanden die Braumeister nur wenig. Während ihrer Arbeit verließen sie sich auf ihre Sinne. So war es in kleineren Haus- und Landbrauereien nicht unüblich, dass die Brauer die Temperatur der abkühlenden Würze in den Pfannen schätzten, indem sie ihren Arm bis zum Ellbogen hineinschoben (Struve 1893: 50). Die Bayerischen Beamten vertrauten auf den Geschmackssinn von Bierkiesern, um die Qualität des Biers zu prüfen, aber auch die Einhaltung der Verordnungen zu kontrollieren: Sie rekrutierten Männer, die bei einer öffentlichen Verkostung die Einhaltungen der Braubestimmungen bezeugten (Gerstner 1859: 255; Hayduck 1925; Kaiser 1835: 666).

### Ballings saccharometrische Bierprobe

Währenddessen änderten sich jedoch langsam die Grundlagen des Braugewerbes. In den 1840er Jahren entwickelte Carl J. N. Balling (1805–1868), Professor für Chemie am Prager Polytechnikum, eine neue Messmethode für die amtliche Bierprobe, die rasch Verbreitung fand. Das Verfahren besaß den Vorzug, dass es genaue Angaben zum Alkohol- und Extraktgehalt eines Biers zuließ (Balling 1843). Der Extraktgehalt gab Auskunft über den Anteil an Stammwürze, die einem gebrauten Bier zugrunde lag. Seine Messung war auch deshalb von Interesse, weil er Rückschlüsse auf die Herstellung des Getränks erlaubte, die in Bayern durch die „Biertaxe“ von 1811 reguliert war.

Im Wesentlichen wurde bei Ballings Methode die Dichte der Flüssigkeiten überprüft. Der Böhme passte hierfür die Skalen eines Saccharometers (Senkspindel) an, mit dem ursprünglich die Konzentration von Zuckerlösungen bestimmt wurde (Balling 1865: 18ff.). Für die saccharometrische Bierprobe nach Balling werden zwei Messungen mit der Senkspindel vorgenommen. Das Gerät besteht aus einem zylinderförmigen Glasrohr. Nach dem Eintauchen zeigt ein beweglicher Schwimmkörper das spezifische Gewicht der Flüssigkeit auf einer Skala an (Hayduck 1925: 42). Bei der ersten Messung wird die Anzeige für das frische Bier abgelesen, aus dem durch Schütteln alle Kohlensäure entfernt wurde. Balling spricht vom scheinbaren Extraktgehalt, da ein Teil des ursprünglichen Extrakts in Alkohol umgewandelt ist. In einem zweiten Schritt wird Bier aus derselben Probe erhitzt, bis der vorhandene Alkohol verdampft ist. Nachdem die verdunstete Menge durch Wasser ersetzt ist, wird der wirkliche Extraktgehalt bestimmt (Balling 1855). Die beiden Messungen müssen hierbei bei einer festen Temperatur vorgenommen werden. Als Richtwert verwendete Balling 14° Réaumur, wobei er auch Tabellen angibt, die eine Umrechnung für andere Temperaturen erlauben.

Seine saccharometrische Bierprobe fußte auf einer Theorie, die Balling in den Jahren 1840 bis 1843 entwickelt hat (Balling 1865: 177). In seinen Augen bildete die „Attenuationslehre“ den „mathematischen Theil“ der chemischen Gärungslehre (Balling 1848: 2). Während seiner Untersuchungen am Prager Polytechnikum war Balling auf eine proportionale Beziehung zwischen dem scheinbaren und dem wirklichen Extraktgehalt des Biers und dem ursprünglichen Extraktgehalt der Würze gestoßen. In langjähriger Arbeit führte er Versuche mit Stammwürzen durch und protokollierte die Veränderung der Saccharometerwerte während des Gärungsprozesses in Tabellen. Seine Untersuchungen ermöglichten ihm später, über die Messung des scheinbaren und wirklichen Extraktgehalts des frischen Biers auf den ursprünglichen Extraktgehalt der Würze zu schließen (Balling 1865: 243; Balling 1848: 3).

**Abb. 3:**
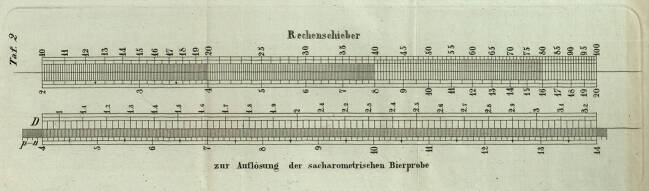
Entwurf eines Rechenschiebers nach Balling (Balling 1848)

Ballings Verfahren besaß nicht zuletzt aufgrund seiner Genauigkeit große Vorteile gegenüber anderen Methoden, die bei der Bierprobe in jener Zeit zur Anwendung kamen. Um den ursprünglichen Extraktgehalt zu ermitteln, mussten allerdings Berechnungen durchgeführt werden. Obwohl diese für heutige Maßstäbe keine größeren Schwierigkeiten bereithielten, waren viele Braumeister mit der Aufgabe überfordert (Balling 1848: 3). Balling präsentierte deswegen im Jahr 1848 neben Tabellen auch die Entwurfszeichnung eines Rechenschiebers, der eine grafische Auflösung der Aufgabe gestattet (Abb. 3) (Balling 1848).

### Vom Extraktgehalt zur -ausbeute

Ballings Tabellen für die saccharometrische Bierprobe fanden große Verbreitung. Mit der zunehmenden Verwissenschaftlichung des Brauereigewerbes in den 1870er Jahren fand sie auch im zunehmenden Maße Anwendung bei der Qualitätskontrolle innerhalb der Brauereien. In größeren Brauereien wurde sie genutzt, um die Qualität des Malzes zu prüfen (Thausing 1882: 501). Zur Probe wurde unter Laborbedingungen aus einer festen Menge Malz durch Maischen Würze hergestellt. Anschließend wurde dann das Gesamtgewicht des gewonnen Extrakts über Ballings Methode ermittelt. Das Verhältnis von Extraktgewicht und verwendeter Malzschüttung – die sogenannte Extraktausbeute – galt hierbei als Maß für die Güte des Rohstoffs.

In seinem Lehrbuch *Die Theorie und Praxis der Malzbereitung und Bierfabrikation* von 1877 erläutert Julius Thausing das Verfahren. Er schildert auch, wie sich die Ausbeuterechnung verwenden lässt, um die Qualität des Maisch- und Sudverfahrens in den Brauereien zu beurteilen. Dafür wird die reale Extraktausbeute, die während des Betriebs im Sudhaus erzielt wird, mit der Ausbeute aus dem Labor verglichen. Der Vergleich werfe Thausing zufolge Licht auf die Arbeitsweise in der Brauerei: „Hat man früher eine Extraction im Kleinen ausgeführt, so lässt ein Vergleich der hierbei und der im Grossen gewonnenen Ausbeute beurtheilen, wie gearbeitet wurde.“ (Thausing 1877: 409)

Die Bayerischen Brauereien besaßen ein großes Interesse daran, die Extraktausbeute zu steigern. Seit 1807 legte der Königliche Hof seiner Besteuerung des Biers die verwendete Menge Malz zugrunde. Für jeden Metzen Malz, den sie verwendeten, mussten die Brauer einen festen Betrag abführen (Struve 1893: 97). Im Lauf des Jahrhunderts wurde die Steuer mehrfach angehoben. Im Jahr 1879 erfolgte dann eine massive Erhöhung um 50 Prozent von 4 auf 6 Mark pro Hektoliter (Hayduck 1925: 312). Diese Abgabe hatte einen hohen Anteil an den Kosten, die bei der Bierherstellung anfielen. Überdies stellte sie lange Zeit den Großteil des Steueraufkommens von Bayern dar (Struve 1893: 29).

Die Erhöhung des Malzaufschlags brachte viele Bayerische Landbrauereien, die mit den großen Brauereien aus München um die Kundschaft konkurrierten, weiter in Bedrängnis. Die Braumeister sannen daher nach Wegen, den Verbrauch des Rohstoffs zu vermindern. Nach der Erhöhung der Abgabe häuften sich die Klagen gegen Brauer, die für das Malz Surrogate verwendeten.^8^ Im Jahr 1884 entbrannte schließlich eine Debatte über die Notlage der Brauer (Holzner 1884; Ratzinger 1884).

Georg Holzner, Professor an der landwirtschaftlichen Schule in Weihenstephan während der Jahre 1869 bis 1892, führte die Misere der kleinen Brauereien auf eine unzureichende Extraktausbeute zurück. Zur Verbesserung der Ausbeuteraten empfahl er unter anderem eine Verbesserung der technischen Einrichtungen. Georg J. Ratzinger (1844–1899), seinerzeit Kaplan in Landshut, widersprach Holzner. Die besseren Extraktausbeuten der Großbrauereien erklärte er mit den Vorteilen, die sie bei der Gerstenbeschaffung besaßen. Während die Familienbetriebe auf dem Land die Rohstoffe für ihre Arbeit aus ihrem unmittelbaren Umfeld bezogen, konnten Erstere Getreide in besserer Qualität auf anderen Märkten beziehen (Ratzinger 1884).

Wie die Diskussion zeigt, entwickelte sich die Extraktausbeute von einem Gütemaß für Malz zu einem universellen Maßstab für die Arbeitseffizienz in den Brauereien. Brauer tauschten sich in Fachzeitschriften über die richtige Ermittlung und die möglichen Ursachen schlechter Ausbeuteraten aus. Autoren unterbreiteten indessen Vorschläge, wie sich die Ausbeute steigern ließe. Unter anderem empfahlen sie eine feinere Schrotung des Malzes, die Verwendung höherwertiger Gerste und die Verlängerung des Maischens. Gegen Ende des Jahrhunderts war die Malzausbeute schließlich maßgeblicher Prüfstein für die rationelle Betriebsführung der Brauereien (Hayduck 1925: 286ff.).

### Ausbeuterechnung nach Windisch

Wilhelm Windisch (1860–1944) widmete dem Thema nach 1890 mehrere Artikel, die in der *Wochenzeitschrift für Brauerei *erschienen sind. Aus dem Taunus stammend, war Windisch 1885 an die neu gegründete Versuchs- und Lehranstalt für Brauerei in Berlin gekommen, wo er bald in der Redaktion der Institutszeitschrift mitwirkte (Hayduck 1925: 425). In einem frühen Artikel widmet er sich dem Thema der Extraktausbeute (Windisch 1902).

Nach seiner Überzeugung habe die Arbeit des Berliner Instituts dazu beigetragen, die Arbeit in den Brauereien zu verbessern:

„Nicht nur, daß die Brauereien in ihrer Allgemeinheit vermehrten Anlaß genommen haben, die Ausbeuten, so wie es zu den Gepflogenheiten einer guten Betriebsführung gehört, regelmäßiger und genauer zu kontrollieren, sondern es haben sich im letzten Jahrzehnt die Ausbeuten in unseren norddeutschen Brauereien ganz erheblich gebessert und sind zum Theil auf eine glänzende Höhe gebracht worden“. (Ebd.: 123)

In seinem Artikel übt Windisch jedoch Kritik an der üblichen Berechnung der Extraktausbeute, die bei der Kontrolle in den Brauereien zur Anwendung kam. Nach seiner Auffassung würde die Kontraktion der Würze beim Abkühlen in der Pfanne nur auf ungenügende Weise berücksichtigt werden. Deswegen schlägt er die Verwendung eines variablen Faktors in Abhängigkeit von der Temperatur vor. Für die Berechnung gibt er dann die Formel

$$F\cdot \frac{hl}{Ztr}$$

an (ebd.: 144). Im Zähler steht hier die Menge an Pfannenwürze in Hektolitern, die nach der Kontraktion auf eine feste Temperatur (14 Grad Réaumur) und nach der Reduktion der Kühlgeläger verbleiben würde, während im Nenner die Malzschüttung in Zentnern einzusetzen ist. Der Faktor F wird aus den abgelesenen Saccharometerwerten über eine Tabelle ermittelt.

**Abb. 4:**
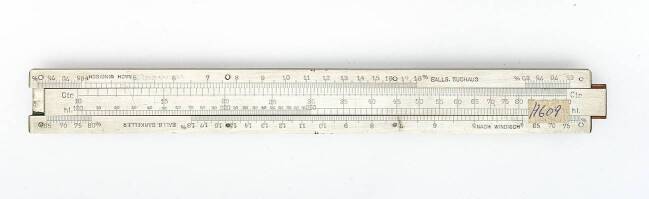
Brauereirechenschieber nach Windisch, 1914 (Deutsches Museum München, Inv.-Nr. 2004-3217)

Auf Grundlage dieser Formel gab die Versuchs- und Lehranstalt für Brauerei 1908 einen Rechenschieber zur Berechnung der Ausbeuteraten heraus (Huß 1912). Um die Handhabung zu vereinfachen, wurde jedoch ein fester Kontraktionsfaktor von 0,96 verwendet.^9^ In der Sammlung des Deutschen Museums befindet sich das Exemplar einer späteren Version des Geräts, das Dennert & Pape – vermutlich im Auftrag – produziert hat (Abb. 4). Ein Aufkleber auf der Rückseite legt das Datum 1914 für die Veröffentlichung nahe. Auf der Zunge sind zwei Skalen für die Malzausschüttung in Zentner und die Menge der Würze in Hektoliter untergebracht. Des Weiteren befinden sich auf dem Rahmen unten und oben jeweils mittig Skalen für die Saccharometeranzeige in Prozentwerten von 4 bis 18. Die Teilung folgt hierbei jedoch den sogenannten Ausbeutefaktoren. Unten und oben sind außerdem Skalen für die Gärkeller- und Sudhausausbeute in Prozentwerten von 65 bis 80 zu sehen. Ein Rechenbeispiel verdeutlicht die Nutzung des Instruments. Zuerst wird der Schieber herausgezogen, bis die verwendete Schüttung in Übereinstimmung mit der ermittelten Saccharometeranzeige ist. Nun lässt sich unterhalb der gemessenen Hektoliterzahl an Würze die Sudhausausbeute ablesen. Der Rechenschieber ist in Abb. 4 gerade für 35 Zentner Schüttung, einem Saccharometerwert von 13 Balling-Prozente und 100 Hektoliter Würze eingestellt. Unten links lässt sich eine Sudhausausbeute von circa 75 ablesen. Die Rechnung mit Windischs Formel liefert 0,7492, wobei für den Ausbeutefaktor F der Wert 23,23 zu nehmen ist.

Die Ausbeuterechnung im Brauwesen lässt erkennen, welche Bedeutung spezifische Kennziffern bei der Durchsetzung rationeller Arbeitsweisen im 19. Jahrhundert besaßen. Während der Diskussionen über die ökonomische Lage der Brauereien in den 1880er Jahren wurde die Extraktausbeute zu einer zentralen Bezugsgröße. Ursprünglich von Carl J. N. Balling im Rahmen seiner mathematischen „Attenuationslehre“ als Qualitätsmaß für Bier eingeführt, entwickelte sich aus dem Extraktgehalt im Lauf der Zeit ein Richtwert für die Effizienz des Produktionsprozesses. Rechenschieber waren ein entscheidendes Mittel, um die Brauer mit den Methoden vertraut zu machen.

## Schiffsdimensionierung

Zu Beginn des 20. Jahrhunderts erlebte der Schiffsbau in Deutschland einen Boom. Im Zuge des Flottenbauprogramms bestellte die Reichsmarine in großer Stückzahl Kriegsschiffe. Aber auch die zivile Schifffahrt füllte die Auftragsbücher der Werften. Die gesteigerte Nachfrage während dieser Zeit veränderte den Schiffsbau – darunter auch die Arbeit der Ingenieure in den Konstruktions- und Kalkulationsbüros. Der Bau neuer und größerer Schiffstypen erforderte neue Verfahren in der Planung und Veranschlagung.

Die Hauptschwierigkeiten bei der Neukonstruktion eines Schiffes lagen in der Dimensionierung. Üblicherweise gab der Auftraggeber wesentliche Leistungsparameter (Tragfähigkeit, mittlere Geschwindigkeit etc.) vor, die das fertige Schiff erfüllen sollte.

Der Ingenieur legte dann den Hauptriss des Schiffes fest, wobei die Planung gewissermaßen von außen nach innen erfolgte. Zuerst wurden die groben Abmessungen des Schiffsrumpfes bestimmt, bevor die Form gezeichnet wurde. Bei Dampfschiffen musste außerdem die benötigte Motorleistung veranschlagt werden. Den Konstrukteuren standen zur Lösung dieser Aufgaben verschiedene Formeln zur Verfügung, die sich in der Praxis bewährt hatten. Sie gestatteten eine erste Abschätzung der gesuchten Maße. Um für eine große Klasse verschiedener Schiffstypen zu gelten, enthielten die Formeln allerdings Parameter, die sich nach der Form richteten. Es blieb die Aufgabe des Konstrukteurs, diese variablen Größen auf Grundlage seiner Erfahrung festzulegen.

### Gewichtsberechnung

Jeder Entwurf beginnt mit der Berechnung der Verdrängung (Deplacement), wie es in *Johows Hilfsbuch für den Schiffsbau* heißt, das um 1900 als Nachschlagewerk in vielen Konstruktionsbüros in Gebrauch war (Johow 1902). Die Zuladung eines Schiffes richtet sich nach der Wassermenge, die es im unbeladenen Zustand verdrängt. Das Deplacement eines Schiffs wiederum entspricht im Wesentlichen seinem Gewicht.

Die genaue Bestimmung des Schiffsgewichts war eine diffizile Aufgabe. Neben der Abmessung des Rumpfes, der Größe von Aus- und Aufbauten und der Anzahl der Besatzung ging auch die Ausrüstung in die Rechnung mit ein. Bei Dampfschiffen fallen außerdem Kohlen und die Maschine ins Gewicht. Für die Entwurfsplanung wurde das Gewicht über das Produkt der Hauptmaße (Länge L, Breite B und Höhe H des Schiffskörpers) abgeschätzt (ebd.: 278). Über einen Faktor c fand die Eigenart des jeweiligen Schiffstyps hierbei Berücksichtigung. Für einen eisernen „Flussdampfer sehr leichter Bauart mit durchlaufenden Aufbauten (Rheindampfer)“ wären nach dieser Methode Kilogramm für das Gewicht zu veranschlagen, wobei c zwischen 150 und 155 zu wählen war. Für hölzerne Jachten falle c indes in den Bereich 100 bis 125 (ebd.: 279).

Wie es in *Johows Hilfsbuch *heißt, muss bei der Methode große Sorgfalt angewendet werden – vor allem bei der Festsetzung von H. Die Höhe des Schiffes richtete sich auch nach der Art der Aufbauten. Eine feste Regel zur Bestimmung gab es hier nicht. Verlangt der Entwurf eine genaue Gewichtsberechnung, so müssen alle Bauteile ausgemessen und ihre Gewichte in speziellen Listen festgehalten werden. Für das Aufstellen dieser Listen gab es wiederum unzählige Regeln wie die Berücksichtigung der Farbe, der inneren Einrichtung, von Decköffnungen etc., die im Buch beschrieben sind (ebd.: 279).

### Schiffbau-Ingenieur-Stockhusen

In der Sammlung des Deutschen Museums befinden sich mehrere Rechenschieber zur Gewichtsberechnung von Schiffen. Das erste Gerät gelangte unter dem Namen „Schiffbau-Ingenieur-Stockhusen“ mit dem Katalog der Jahre 1905 bis 1906 in den Verkauf.^10^ Der Rechenschieber gehört damit zu den ältesten Sondergeräten im Sortiment der Hamburger Firma Dennert & Pape.

Namensgeber ist der Schiffsbauingenieur Carl Stockhusen (1869–1948). Er hatte als Lehrling bei einer Werft in Elmshorn die Arbeit von der Pike auf gelernt (Anonym 1951). Sein Weg führte ihn 1895 zur Germaniawerft nach Kiel, wo er mit dem Bau von Torpedobooten beauftragt war. Im Rahmen seiner Tätigkeit war er jedoch auch mit der Konstruktion von Segeljachten befasst. Nach weiteren Anstellungen bei verschiedenen Werften übernahm er 1905 den Posten des stellvertretenden Bürochefs der Kieler Howaldtswerke. Stockhusen erwarb während seiner Berufslaufbahn große Erfahrung in der Konstruktion verschiedener Schiffstypen. Außerdem war er Gründungsmitglied der Schiffbautechnischen Gesellschaft. Sein Beitrag zur Verwendung von Laufkränen in modernen Werftanlagen blieb allerdings einer von wenigen Fachartikeln, die der Ingenieur veröffentlichte (Stockhusen 1903).

**Abb. 5:**
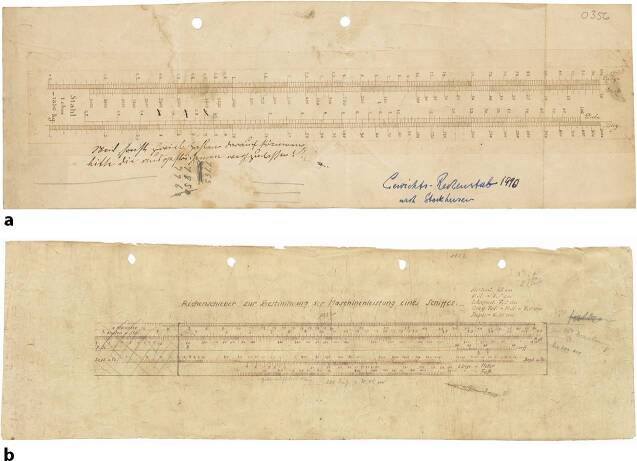
Entwurfszeichnungen für zwei Rechenschieber nach Konstruktion von Carl Stockhusen: (**a**) Gewichtsrechenschieber nach Stockhusen und (**b**) Rechenschieber zur Bestimmung der Maschinenleistung eines Schiffs (DMA Deutsches Museum, München, Archiv. FA 006/0703.)

Im Firmenarchiv von Dennert & Pape am Deutschen Museum befindet sich eine Entwurfszeichnung des Rechenschiebers (Abb. 5). Über drei Skalen wurden die Länge, Breite und Dicke der Stahl- oder Eisenstücke eingestellt, über eine vierte Skala ließ sich hiernach das Gewicht in Kilogramm ablesen. Mit der Vorderseite des Schiebers ließen sich so die Gewichte von Eisenteilen berechnen, während die Rückseite zur Berechnung von Stahlteilen diente. Zur Orientierung ist jeweils das spezifische Gewicht – 7.763 Kilogramm pro Kubikmeter Eisen und 7.850 Kilogramm pro Kubikmeter Stahl – angegeben. In einer Anleitung heißt es: „Da die Handhabung die denkbar einfachste ist, so erfordert der Gebrauch des Gewichtsschiebers nicht erst eine längere praktische Übung wie dies beim gewöhnlichen Rechenschieber unumgänglich nötig ist, sondern er kann sofort von jedem Laien benutzt werden.“^11^ Die Skalenteilung und -beschriftung erleichterten die Bedienung des Gewichtsrechenschiebers nach Stockhusen. Um das Gewicht eines Metallteils zu bestimmen, hätte allerdings ein gewöhnlicher Rechenschieber genügt.

### Maschinendimensionierung nach dem System Stockhusen

**Abb. 6:**
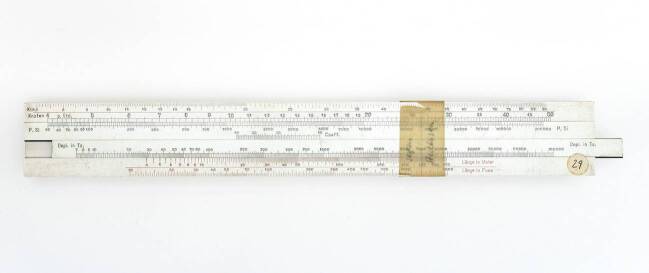
Rechenschieber zur Dimensionierung der Schiffsmaschinen nach dem System Stockhusen (Deutsches Museum München, Inv.-Nr. 2004-2298)

Von Carl Stockhusen stammen noch weitere Rechenschieber, die zur Dimensionierung von Dampfschiffmaschinen dienten. Ein erster Entwurf erschien 1924 unter dem Namen „Schiffbauer“ bei Dennert & Pape. In einer Anleitung, die sich im Nachlass der Firma befindet, wird Stockhusen als Urheber des Entwurfs genannt.^12^ Später erschien ein neuer Rechenschieber unter dem Namen „System Stockhusen“ (Abb. 6). Im Gegensatz zum ersten Entwurf war das Instrument durch und durch auf die Aufgabe der Dimensionierung ausgelegt. In den Unterlagen der Firma befindet sich auch eine Entwurfszeichnung dieses Geräts (Abb. 5).

Ein zentrales Problem beim Entwerfen von Dampfschiffen bestand darin, dass die Leistung der Maschinen veranschlagt werden musste, noch bevor die genaue Form des Schiffsrumpfs feststand. Die Leistung hing jedoch vom Widerstand ab, den das Schiff bei der Fahrt durchs Wasser überwinden musste (Johow 1902: 624). Für die Abschätzung war lange Zeit die englische „Admiralitätsformel“

$$P=\frac{D^{2/3}\cdot V^{3}}{C}$$

in Gebrauch, bei der die Leistung P mithilfe des Deplacements D und der mittleren Geschwindigkeit V ermittelt wird. Der Koeffizient c richtet sich nach dem Typ des projektierten Schiffs. Obgleich die Formel in der Praxis weit verbreitet war, stieß sie immer wieder auf Kritik. Das Verhältnis von Länge zu Breite, das für den Widerstand des Schiffskörpers im Wasser maßgeblich ist, spiegelt sich in dem Ausdruck nicht wider (Atherton & Mellanby 1903: 21). Während der zweiten Hälfte des 19. Jahrhunderts wurden deswegen immer wieder neue Formeln vorgeschlagen, die eine bessere Annäherung an die wirklichen Verhältnisse versprachen (Johow 1902: 633ff.).

Auch Stockhusen beschäftigte sich mit dem Problem der Maschinendimensionierung. In der Anleitung für den ersten Rechenschieber „Schiffbauer“ wird noch beschrieben, wie sich die Aufgabe unter Verwendung der Admiralitätsformel lösen lässt. Als er später ein neues Gerät entwarf, ging Stockhusen einen anderen Weg. Ein Text, der sich im Nachlass der Firma Dennert & Pape befindet und Werbezwecken diente, lässt den Gedanken erkennen, der den Ingenieur hierzu bewegte:

„Wenngleich auch in der neueren Zeit mehrere, zum Teil gute Formeln aufgestellt worden sind, so ist die Berechnung, wenn sie mit gewöhnlichen Mitteln ausgeführt wird, nicht nur sehr zeitraubend, sondern auch von gewissen Erfahrungs-Cöfficienten abhängig. Es wird deshalb zum Projektieren von Neubauten, um zunächst schnell einen Überblick zu haben, immer noch sehr viel die alte Admiralitäts-Formel benutzt, obgleich der Coeff. von 50–450 schwankt, sodass es für jüngere Konstrukteure, die noch nicht über genügend Erfahrung verfügen, schwierig wird, für den in Frage kommenden Schiffstyp den richtigen Coeff. zu wählen“.^13^

Die Wahl der passenden Parameter verlangte Erfahrung. Junge Ingenieure, die ihren Beruf auf den technischen Hochschulen gelernt hatten, stellte die Aufgabe vor Hürden. Die Schwierigkeiten wuchsen mit der Anzahl an Werten, die zur Auswahl standen. Der Rechenschieber „System Stockhusen“ sollte diese diffizilen Entscheidungen erleichtern. Wie aus dem zitierten Text hervorgeht, legte Stockhusen dem Entwurf eine Formel zugrunde, die er „auf Grundlage von Erprobungen einer großen Anzahl von Schiffen verschiedener Typen“ gewonnen hatte.^14^ Welche Formel er allerdings verwendete, teilt er an dieser Stelle nicht mit. Eine Bleistiftnotiz am Rande der Entwurfszeichnung gibt nur grobe Hinweise (Abb. 5).

Im Gegensatz zur Admiralitätsformel berücksichtigt Stockhusens Formel das Verhältnis vom Deplacement zur Schiffslänge. Dies geht auch aus dem Beispiel hervor, das in dem Text vorgerechnet wird. Außerdem ist das Spektrum des Typkoeffizienten c auf den Bereich 1 bis 40 eingeschränkt. In dem Text werden für verschiedene Schiffstypen – von Schleppdampfern und Unterseebooten bis hin zu Zerstörern – jeweils passende Wertebereiche angegeben. Die obere Zunge des Instruments zeigt die Skala für den Koeffizienten (Abb. 6). Auf der unteren Zunge ist das Deplacement verzeichnet, während sich auf dem Rahmen weitere Teilungen für die mittlere Geschwindigkeit in Knoten (oben) sowie für die Länge und Breite des Schiffes (unten) befinden.

### Dietzes „Enni“-Schieber

Ein weiterer Rechenschieber aus der Sammlung des Deutschen Museums stammt von Ernst Wilhelm Dietze (1837–1915). Nach seinem Studium an der Polytechnischen Schule in Dresden ging Dietze für einige Jahre nach Schweden, wo er unter anderem an einer Werft in Nyköping Anstellung fand. Zurück in Deutschland, ließ er sich in Leipzig für einige Zeit als Zivilingenieur nieder (Lehmann 1999). In diesen Jahren begann er seine ersten theoretischen Arbeiten zum Schiffbau zu verfassen. Im Jahr 1863 nahm er dann eine Stelle als Ingenieur bei der Sachsenberg-Werft Roßlau an (Düntzsch & Hinsch 1997: 24). In den folgenden Jahren entwarf Dietze verschiedene Boote für die Flussschifffahrt auf der Elbe. Er zeigte großes Interesse an technischen Fragen der Schiffskonstruktion. Als einer der ersten deutschen Ingenieure stellte er umfangreiche Berechnungen zur Längenfestigkeit flacher und langer Schleppdampfer an (Lehmann 1999: 105). Größeres Ansehen erwarb er sich auch durch die Konstruktion eines optimierten Schaufelrads für Flussschiffe.

**Abb. 7:**
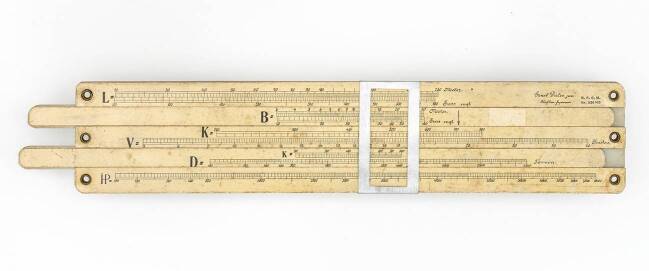
Schiffsrechenschieber nach Wilhelm Dietze (Deutsches Museum München, Inv.-Nr. 2004-1705)

Dietze beschäftigte sich mit den Berechnungen, die im Schiffbau nötig waren. Für einen Artikel in der *Zeitschrift des Vereins Deutscher Ingenieure* entwarf er 1887 mehrere Nomogramme zur grafischen Ermittlung des Schiffswiderstands (Dietze 1887). Im Deutschen Museum befindet sich außerdem ein Rechenschieber, mit dem sich die indizierte Motorleistung für verschiedene Schiffstypen berechnen ließ. Angaben auf der Vorderseite des Geräts legen nahe, dass der Entwurf von Ernst Dietzes Sohn stammt^15^ (Abb. 7). Nicht sicher ist auch, von wem das Gerät hergestellt wurde. Selbst eine Werbebroschüre, die sich im Museum befindet, gibt hierauf keine Hinweise. Nach der Bauart zu urteilen, wurde das Gerät nicht von Dennert & Pape produziert. In der Reklameschrift wird der Rechenschieber unter dem Namen „Enni-Schieber“ für vier Mark angeboten. Der Preis spricht dafür, dass der Verkauf für die Zeit nach Einführung der Reichsmarkt 1924 geplant war.

Auf dem Rechenschieber befinden sich sieben Skalen, die sich auf den Rahmen und zwei Zungen verteilen. Im oberen Bereich finden sich Teilungen für die Schiffslänge und -breite (L und B), in der unteren Hälfte Skalen für das Deplacement D, die indizierte Pferdestärke und die maximale Geschwindigkeit V. Überdies befinden sich zwei Teilungen auf dem Rechenschieber, die einem Formkoeffizienten K vorbehalten sind. Beim „Enni-Schieber“ handelt es sich hierbei jedoch um eine Hilfsgröße. Sie wird nicht durch den Ingenieur als Erfahrungswert festgelegt, sondern über die Länge und Breite des Schiffes berechnet. Nachdem der Koeffizient kalkuliert wurde, wird in einem zweiten Schritt unter Vorgabe der maximalen Geschwindigkeit V die benötigte Pferdestärke ermittelt.

In der Broschüre wird das Vorgehen durch mehrere Beispiele erläutert: Für den Schnelldampfer „Kaiser Wilhelm II“ sei L = 206,65 m, B = 21,94 m, D = 21.000 t. Nachdem die Werte für Länge und Breite durch das Verschieben der oberen Zunge in Deckung gebracht worden sind, lässt sich unter dem Pfeil der Koeffizient K = 750 ablesen. Hiernach wird nun die untere Zunge verschoben, bis der ermittelte Wert K = 750 auf der anderen Skala gegenüber von der vorgegebenen Geschwindigkeit V = 23,58 kn liegt. An der Stelle des gegebenen Deplacements D = 21.000 t erscheint dann die gesuchte Pferdestärke 43.800 hp.

Interessanterweise wird die dem Rechenschieber zugrunde liegende Formel nicht mitgeteilt. Was die Anwendung betrifft, werden hierfür nur wenige Einschränkungen gemacht. Obwohl die Werte des Schiebers für „gewöhnliche Handelsschiffe“ gelten, sei das Instrument auch für die Konstruktion anderer Schiffstypen zweckdienlich, wie es in der Werbeschrift heißt. Hierfür müssten prozentuale Anteile der abgelesenen Pferdestärke verwendet werden, wobei sich die Größe der Anteile nach dem Typ richtet. Zwar werden einige Prozentsätze angegeben, zugleich heißt es jedoch, dass der Umrechnungsfaktor von „jedem Fachmann aus eigener Praxis leicht festzustellen“ sei. Demnach kam es am Ende doch auf die Erfahrung des Ingenieurs bei der Anpassung der Ergebnisse an.

Die passende Dimensionierung der Maschine war ein mathematisches Kernproblem beim Schiffbau. Die große Anzahl unterschiedlicher Schiffstypen, die erheblich in Größe, Aufbau und Form variierten, stand der Verwendung einer allgemeingültigen Formel im Wege. Koeffizienten in den Formeln sollten den technischen Unterschieden gerecht werden. Dem Ingenieur fiel hierbei die Aufgabe zu, auf Grundlage seiner Berufserfahrung einen passenden Wert zu wählen. Im Design der Formeln – aber auch der Rechenschieber – spiegeln sich widerstrebende Ziele wider. Einerseits sollten sie den technischen Spezifika des jeweiligen Schiffstyps gerecht werden, andererseits eine breite Klasse von Fällen miteinschließen und leicht zu verwenden sein.

## Zusammenfassung

Spezialrechenschieber erleichterten die Anwendung mathematischer Methoden. In den Jahren der Hochindustrialisierung leisteten sie dadurch einen wesentlichen Beitrag zur Substitution handwerklicher Praktiken durch wissenschaftliche Methoden (1.). Mit den Rechenschiebern ließen sich in Tätigkeitsfeldern, in denen Erfahrung einen hohen Stellenwert besaß, exakte Verfahrens- und Arbeitsweisen durchsetzen. Die Konstruktion passender Maße, die hierbei eine entscheidende Rolle spielte, war nicht zuletzt eine mathematische Aufgabe, wie Ballings saccharometrische Bierprobe zeigt. Die Anwendung quantifizierender Messverfahren war ein erster Schritt für die Konstruktion ökonomischer Kennziffern (Extraktausbeute), mit denen sich der Produktionsprozess überwachen ließ (2.). Die Geräte trugen zur Standardisierung von Arbeitsweisen bei. Die Ausübung professioneller Tätigkeiten wurde durch die Einführung spezieller Rechenschieber Regeln unterworfen. Für Sachentscheidungen – wie die Einstellung von Maschinen – ließen sich allgemeingültige Normen aufstellen. Die Geräte waren ein Instrument, um die Ausübung sensibler Tätigkeiten in vielen Bereichen zu formalisieren. Dadurch wurde nicht nur die Kontrolle des Managements über die Vorgänge in der Werkshalle ausgedehnt, sondern auch die Arbeit vereinfacht (3.). Dieser Aspekt zeigt sich bei der Schiffskonstruktion auf den Werften. Die Spezialrechenschieber erleichterten die Tätigkeit der Ingenieure. Sie machten Rechnungen überflüssig, vereinfachten jedoch auch die Ausführung, indem sie Aufgaben wie die Maschinendimensionierung auf die Festlegung spezieller Erfahrungskoeffizienten herunterbrachen. Bei der Projektierung eines neuen Schiffs musste der Konstrukteur nicht erst überlegen, wie er die vorhandenen Methoden an sein Problem anpasst. Der Rechenschieber reduzierte seine Aufgabe darauf, auf Grundlage seiner Erfahrung eine passende Zahl innerhalb eines präskriptiven Wertebereichs zu bestimmen.

Die vorangegangenen Fallbeispiele haben nicht nur Licht auf die sozioökonomischen Aspekte der Integration mathematischer Methoden in Arbeitsgebiete geworfen. Im Einzelnen haben sie auch technische Probleme aufgezeigt, die der breiten Anwendung im Weg standen. Die Nützlichkeit von Rechenschiebern zur Kalkulation des optimalen Vorschubs wurde durch die fehlende Standardisierung bei den Drehbänken begrenzt. Die Ergebnisse, die durch Untersuchungen an speziellen Maschinen gewonnen wurden, ließen sich nicht ohne Weiteres auf andere Geräte übertragen. Die Verbreitung mathematischer Methoden hing – wie dieses Beispiel erhellt – auch von technologischen Voraussetzungen ab.

Spezialrechenschieber bilden einen vielversprechenden Ausgangspunkt für historische Studien in der Wirtschafts‑, Technik- und Mathematikgeschichte. Die Geschichten der Objekte gewähren Einblicke in die spezifischen Probleme, die mit der Anwendung mathematischer Methoden in technische Arbeitsgebiete verbunden waren. Sie lassen aber auch wirtschaftliche, technische und soziale Faktoren erkennen, die hieran Anteil hatten. Dementsprechend sind sie nicht nur für eine Historiografie der Mathematik von Interesse, die die gesellschaftlichen Verflechtungen der Wissenschaft ins Blickfeld rückt. Die Geräte geben auch Auskunft über die lokalen Aspekte, die bei der Rationalisierung von Gewerben und Disziplinen im 19. und 20. Jahrhundert mitwirkten.

## Danksagung

Für das Forschungsprojekt gewährte mir das Deutsche Museum in München ein Scholar-in-Residence-Stipendium. Vor Ort halfen mir verschiedene Personen bei der Umsetzung des Projektes, denen ich meinen Dank aussprechen möchte. Namentlich möchte ich Katja Rasch, Kuratorin für Mathematik am Museum, herzlich für ihre Unterstützung danken.

## Footnotes

1 Vom Umfang dieser Tätigkeit legen die Firmenunterlagen Zeugnis ab: Kalkulationsverzeichnis 1937–1942. DMA Deutsches Museum, München, Archiv. FA 006/0029.

2 Die Jahreszahl ist einer Liste mit Angaben zur Veröffentlichung einzelner Rechenschieber entnommen, die vom letzten Inhaber stammt. DMA Deutsches Museum, München, Archiv. FA 006/0078.

3 Betriebsrechenschieber System Friedrich und Hippler (Werbeschrift). DMA Deutsches Museum, München, Archiv. FA 006/0705.

4 Preiskatalog. DMA Deutsches Museum, München, Archiv. FA 006/0538.

5 Bei vielen von Taylors Team entworfenen Rechenschiebern konnten die Zungen ausgewechselt werden. Dadurch ließen sie sich für verschiedene Maschinen verwenden.

6 Das genaue Jahr lässt sich nicht ermitteln.

7 Firmenschrift. DMA Deutsches Museum, München, Archiv. FA 006/0540.

8 Als Malzsurrogate wurden häufig Traubenzucker und Zuckersirup verwendet (Gerstner 1859: 249).

9 Die Berechnung wird in einem Bier-Lexikon erläutert, das im Umfeld des Instituts erschienen ist (Hayduck 1925: 60, 290).

10 Firmenschrift. DMA Deutsches Museum, München, Archiv. FA 006/0532.

11 Die Anleitung findet sich auf einer CD als Beilage zu Kühn (2004).

12 Rechenschieber zur Bestimmung der Maschinenleistung eines Schiffes. DMA Deutsches Museum, München, Archiv. FA 006/0700.

13 Rechenschieber zur Bestimmung der Maschinenleistung eines Schiffes. DMA Deutsches Museum, München, Archiv. FA 006/0700.

14 Rechenschieber zur Bestimmung der Maschinenleistung eines Schiffes. DMA Deutsches Museum, München, Archiv. FA 006/0700.

15 Nach einer Nummer auf dem Holzlineal wurde das Gebrauchsmuster außerdem im Jahr 1922 eingetragen. Zu diesem Zeitpunkt war Ernst Dietze Senior bereits verstorben.
